# Boundary quantitative characterization of the top-coal limit equilibrium zone in fully mechanized top-coal caving stope along the strike direction of working face

**DOI:** 10.1038/s41598-024-65655-4

**Published:** 2024-06-24

**Authors:** Ding Lang, Xiaobo Wu, Yongping Wu, Panshi Xie, Zhuangzhuang Yan, Shuaiming Chen

**Affiliations:** 1 School of Energy and Mining Engineering, Xi’an University of Science and Technology, Xi’an, 710054 China; 2 Key Laboratory of Western Mines and Hazard Prevention, Ministry of Education of China, Xi’an, 710054 China

**Keywords:** Fully mechanized top-coal caving mining, Limit equilibrium zone, Mining-induced stress, Quantitative characterization, Engineering, Geology

## Abstract

In the process of fully mechanized top-coal caving mining, the top-coal is affected by mining-induced stress, and the stress varies along the strike direction of working face, so the boundary position of its entering the limit equilibrium state changes accordingly. The determination of the boundary along the strike direction of working face can provide scientific guidance for the stability control of support-surrounding rock in fully mechanized top-coal caving face. Using the research methods of theoretical analysis, physical similarity simulation experiment and numerical simulation experiment, the stress state analysis model of the boundary position of the top-coal limit equilibrium zone under macro-scale conditions was established, the stress state characterization method of the boundary of the top-coal limit equilibrium zone along the strike direction of working face was given, and the quantitative characterization of the boundary of the top-coal limit equilibrium zone along the strike direction of working face was realized by combining with the mining-induced stress path, and the distance relationship between the boundary of the top-coal limit equilibrium zone and the langwall face along the strike direction of working face was revealed. The results show that after critical mining in fully mechanized top-coal caving face, the distance between the boundary of top-coal limit equilibrium zone and the langwall face along the strike direction of working face presents a relationship of increasing from top to bottom. The distance between the top-coal upper boundary and the langwall face was 2.85 m and the distance between the top-coal lower boundary and the langwall face was 5.39 m. The boundary of top-coal limit equilibrium zone along the strike direction of working face was verified by the top-coal elastic–plastic zone boundary and the boundary of the peak position of front abutment pressure in different layers of top-coal. The results show that the quantitative characterization of the top-coal limit equilibrium zone boundary along the strike direction of working face was reasonable. In order to improve mine production efficiency, optimization measures were put forward for hard coal seam and soft coal seam respectively.

## Introduction

Fully mechanized top-coal caving mining is one of the main methods of thick seam mining^1–5^. Top-coal is the only load transfer medium between support and roof, and its physical and mechanical state is the key factor to determine the contact relationship between support and top-coal. In the process of mining, the top-coal is affected by ground pressure from the starting position in front of the langwall face to the drawing state, and its deformation and failure are gradually cumulative evolution. Therefore, realizing the quantitative characterization of top-coal failure process will help to improve the production efficiency of coal mines. According to different stope conditions, accurate and effective measures can be implemented to improve production efficiency. For example, if the coal seam strength is hard, accurate pre-splitting can be carried out according to the quantitative results of the boundary of the top-coal limit equilibrium zone to ensure the top-coal caving rate.

Experts have done a lot of work on the study of top-coal failure process. According to the deformation law of top-coal^6–8^, the top-coal can be divided into different stages. Through the analysis of the measured data of top-coal displacement^9,10^, the top-coal displacement in front of the langwall face was mainly horizontal displacement, and when it was close to the langwall face, the top-coal displacement was mainly vertical displacement. In fully mechanized top-coal caving mining of extra-thick coal seam^11^, the displacement of upper top-coal was greater than that of middle top-coal, the displacement of middle top-coal was greater than that of lower top-coal, and the displacement of the middle section of the top-coal layer exceeded that of the upper section in mining stage. By analyzing the evolution law of cracks in top-coal under the action of the front abutment pressure^12–14^, the quantitative description of the evolution law of cracks was realized, and the development characteristics of the rack field in top-coal were determined.

The revelation of the crack propagation law of top-coal promotes the research of damage mechanics on the failure process of top-coal. The constitutive equations of damage characteristics in the advancing direction and along the strike direction of working face were established by using macroscopic damage mechanics^15^. The statistical damage constitutive equation of top-coal unit under uniaxial compression was established^16–18^. The damage mechanical model under the action of abutment pressure was established^19,20^. The critical position of top-coal medium state significantly affects the interaction between top-coal and support. The damage mechanism of top-coal under different loads along the inclined direction of working face was revealed. The progressive damage model of top-coal under the path of mining-induced stress was established, and the boundary position relationship of top-coal limit equilibrium zone under different coal seam strength conditions was analyzed^21–24^.

At present, the research mainly studies the failure process of top-coal from three aspects: crack propagation mechanism, deformation law and damage degree of top-coal. Qualitative description based on the characteristics of ground pressure behavior of top-coal or quantitative discussion based on rock strength theory. There is little research on top-coal along the strike direction of working face, and there is even less research on the shape and quantification of the boundary of top-coal limit equilibrium zone in fully mechanized top-coal caving face. Based on elasticity and Mohr–Coulomb criterion, an analytical model of stress state at the boundary of top-coal limit equilibrium zone under macro-scale conditions is established, and the stress state characterization of the boundary of top-coal limit equilibrium zone along strike is obtained. Combined with mining-induced stress, the quantitative characterization of the boundary of top-coal limit equilibrium zone along the strike direction of working face under specific conditions is realized, and the spatial relationship between the boundary of top-coal limit equilibrium zone and langwall face along the strike direction of working face is revealed. The research results are helpful to improve the production efficiency of coal mine, provide scientific guidance for the stability control of “support-surrounding rock” in fully mechanized top-coal caving face and provide theoretical reference for realizing the intelligence of fully mechanized top-coal caving face.

## Generation mechanism of the top-coal limit equilibrium state

**Figure 1:**
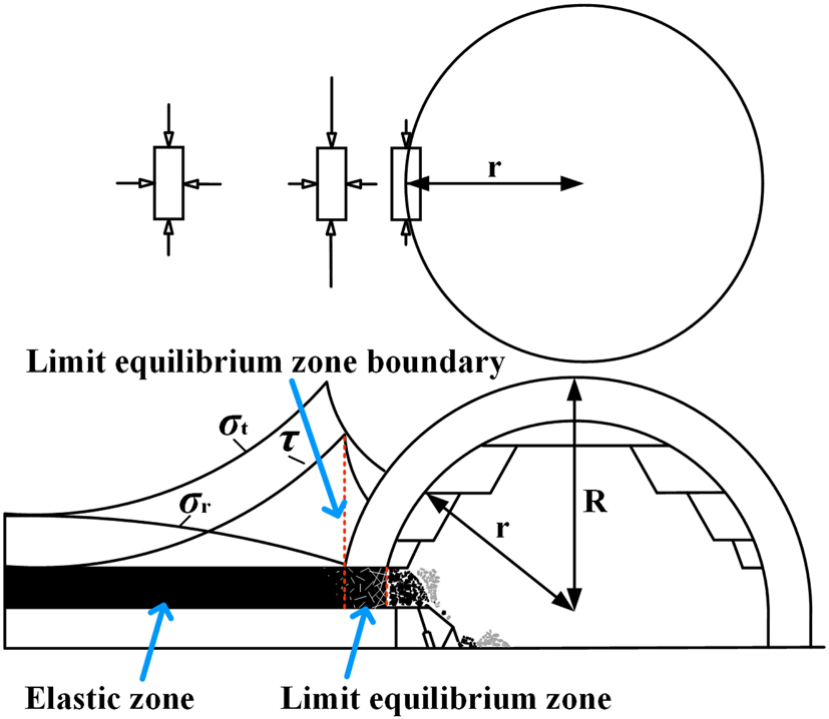
Limit equilibrium state of coal and rock mass in fully mechanized top-coal caving stope.

In the mining process of working face, with the increase of advancing distance, the roof will sink and break continuously. The stress distribution within the stope will be altered in response to changes in the overburden structure, and the caving arch of overlying strata will continue to expand. At the boundary of caving arch, the rock mass is always in a unidirectional stress state, With the transfer to the deep surrounding rock, the unidirectional stress state of the rock mass gradually changes to the three-directional stress state, and the rock mass will not be disturbed by mining and will be in an elastic state. As shown in Fig. 1, the rock mass outside the boundary with radius *R* is undamaged, and its state can be characterized by elastic mechanics. The hole with radius *r* is the boundary where the stress state of the rock mass changes from three-way stress to unidirectional stress. Therefore, along the advancing direction of the working face, the top-coal clamped by unloading boundary and elastic boundary of surrounding rock in stope is in a limit equilibrium state^25^. *σ*_t_, *σ*_r_, and *τ* are tangential stress, radial stress and shear stress of rock mass around the stope, respectively, *R* is the outer radius of the limit equilibrium circle of the hole, and *r* is the inner radius of the limit equilibrium circle of the hole.

## Characterization of stress state at the boundary of the top-coal limit equilibrium zone

Top-coal is the only medium clamped between the support and the roof, and its mechanical state changes gradually under mining stress. Under the influence of mining-induced stress, top-coal goes through a complicated stress loading process from the original state to the loose state. During this process, shear failure occurs inside the coal body, and gradually expands with the shear failure surface. Because the unexposed coal behind the support has a certain limiting effect on the surrounding coal and rock mass, the fracture expansion and mutual penetration in the coal are limited by space. So that there is still some extrusion and friction between the fractured unit blocks, and the coal still maintains a certain continuity and bearing capacity on the macro level, that is, it is in a fragile equilibrium state, which is called the limit equilibrium state. The distance span of the top-coal limit equilibrium state along the strike is called the top-coal limit equilibrium zone.

**Figure 2:**
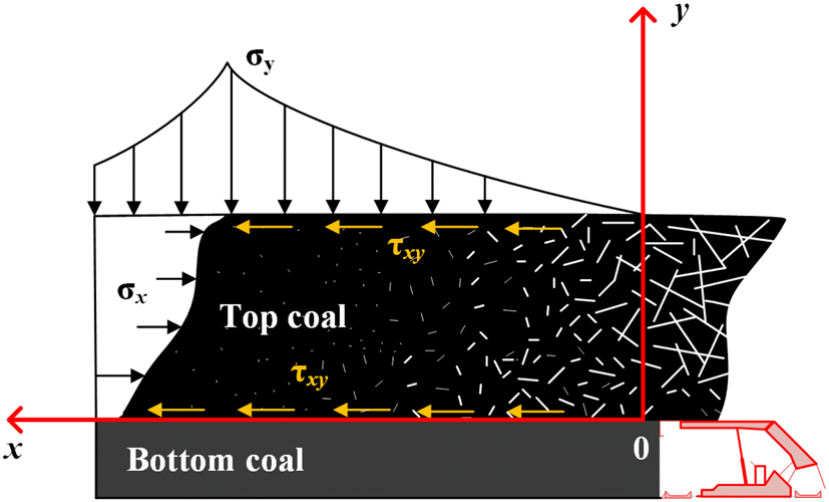
Mechanical model of top-coal limit equilibrium zone.

The strength of coal seam roof and floor is generally higher than that of coal seam. When the coal seam is not disturbed by mining and constrained by boundary conditions, the coal seam is under pressure for a long time. In the fully mechanized top-coal caving face, with the top-coal caving from the back of the support, the constraint of the coal seam is relieved, so the top-coal will slide towards the goaf under the extrusion of the roof and floor. Based on the above conditions, the mechanical model of top-coal is established, as shown in Fig. 2. The top-coal is subjected to vertical stress *σ*_*y*_, *σ*_*x*_ is the lateral stress of the top-coal, and *τ*_*xy*_ is the friction force given to it by the upper and lower boundaries when the top-coal slides outward.

It is assumed that there are slip surfaces between top-coal and roof, top-coal and bottom coal in the process of coal caving in working face:

1 $$\tau_{xy} = - \;(\sigma_{y} \tan \varphi_{0} + c_{0} )$$

where *φ*_0_ is the internal friction angle of coal seam, *c*_0_ is cohesion of coal seam.

When the top-coal is extruded from the middle of the roof and bottom coal, the top-coal stress should satisfy the stress limit equilibrium condition, and the top-coal stress in the region should satisfy the stress differential equilibrium equation. Without considering the volume force, the basic equation for solving the interfacial stress in the limit equilibrium zone is:

2 $$\frac{{\partial \sigma_{x} }}{\partial x} + \frac{{\partial \tau_{xy} }}{\partial y} = 0$$

3 $$\frac{{\partial \tau_{xy} }}{\partial x} + \frac{{\partial \sigma_{y} }}{\partial y} = 0$$

The Eqs. (2) and (3) are combined:

4 $$- \frac{{\partial \sigma_{y} }}{\partial x}\tan \varphi_{0} + \frac{{\partial \sigma_{y} }}{\partial y} = 0$$

It can be seen from Eq. (4) that the partial differential equation is homogeneous, and the homogeneous partial differential equation can express the solved unknown function as the product of multiple unknown unary functions. After substituting into the original partial differential equation, the partial differential equation can be decomposed into multiple uncoupled ordinary differential equations, and the number of ordinary differential equations is the number of independent variables. Let the equation be:

5 $$\sigma_{y} = f(x)\;g(y) + W$$

where *W* is a constant term. Bring Eq. (5) into Eq. (4):

6 $$- f^{\prime}(x)g(y)\;\tan \varphi + f(x)g^{\prime}(y){ = }0$$

7 $$\frac{{f^{\prime}(x)}}{f(x)}\;\tan \varphi { = }\frac{{g^{\prime}(y)}}{g(y)}$$

According to Eq. (7), the two sides of the equation are only functions about x or y respectively, and it can be assumed that both sides are equal to the same constant *W*:

8 $$\left\{ \begin{gathered} \frac{{f^{\prime}(x)}}{f(x)}\;\tan \varphi = W \hfill \\ \frac{{g^{\prime}(y)}}{g(y)} = W \hfill \\ \end{gathered} \right.$$

Solve Eq. (8):

9 $$\left\{ \begin{gathered} f(x){ = }W^{\prime}_{1} e^{{\frac{W}{\tan \varphi }x}} \hfill \\ g(y) = W^{\prime}_{2} e^{Wy} \hfill \\ \end{gathered} \right.$$

where $W^{\prime}_{1}$ and $W^{\prime}_{2}$ are constant terms. Substitute Eq. (9) into Eq. (5):

10 $$\sigma_{y} = W^{\prime}_{1} \;W^{\prime}_{2} \;e^{Wy} e^{{\frac{Wx}{{\tan \varphi }}}} + W_{1}$$

where *W*_1_ is a constant term. Equation (10) is the functional relationship between vertical stress and coordinates x and y in the limit equilibrium zone. By solving the Eq. (10):

11 $$\sigma_{y} = W^{\prime}_{1} \;W^{\prime}_{2} \;e^{{\frac{W}{\tan \varphi }\left( {y\tan \varphi + x} \right)}} + W_{1}$$

## Distribution law of top-coal stress in fully mechanized top-coal caving face

Based on the above research contents, the relationship between the boundary of the top-coal limit equilibrium zone and the vertical stress *σ*_y_ in fully mechanized top-coal caving face is derived. In order to determine the relevant parameters in the relationship, It is necessary to determine the stress distribution law of top-coal and determine the relevant parameters in combination with the Eq. (10). The 1123 working face of a mine in Gansu Province is a fully mechanized top-coal caving face, and its engineering background is just suitable for this study. Firstly, the state of mining-induced stress stability is determined by the caving state of overlying strata and the compaction of goaf. It is more reasonable to analyze the stress distribution law of top coal when the mining stress is stable.

### Physical similarity simulation experiment parameters and process

Considering the 1123 fully mechanized top-coal caving face of a mine in the Gansu province of China as the research focus, the average thickness of the coal seam considered for the face was 9 m, strike length was 530 m, length along the dip was 100 m, average buried depth was 330 m, designed mining height was 3 m, and caving height was 6 m. Comprehensive mechanized top-coal caving mining technology was employed, and the roof was managed using all available caving methods. Physical and mechanical parameters of coal seam, roof and floor provided by the mine are listed in the Table 1.

**Table 1:** Coal and rock physical and mechanical parameters.

Number	Rock layers	Volume weight (kg/m^3^)	Modulus of elasticity (MPa)	Uniaxial compressive strength (MPa)	Cohesion (MPa)	Internal friction angle (°)
1	Siltstone	2350	6000	16.0	2.8	28
2	Coal	1350	3900	10.0	2.7	23
3	Carbon mudstone	2500	5500	18.0	4.0	23
4	Mudstone	2470	3800	20.0	2.9	30
5	Fine sandstone	2710	5600	34.0	3.9	25
6	Coarse sandstone	2570	4800	28.0	5.0	32
7	Sandstone	2520	4900	36.0	2.9	35
8	Fine sandstone	2710	5600	35.0	3.7	25
9	Conglomerate	2520	4900	29.0	3.0	33
10	Coarse sandstone	2570	4800	28.0	4.6	32

**Figure 3:**
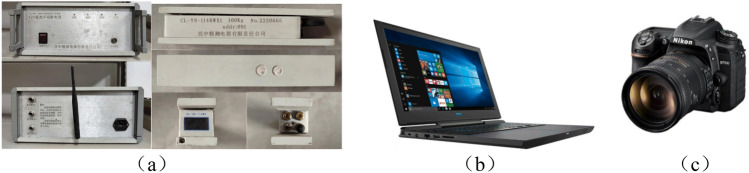
Experimental equipment.

**Figure 4:**
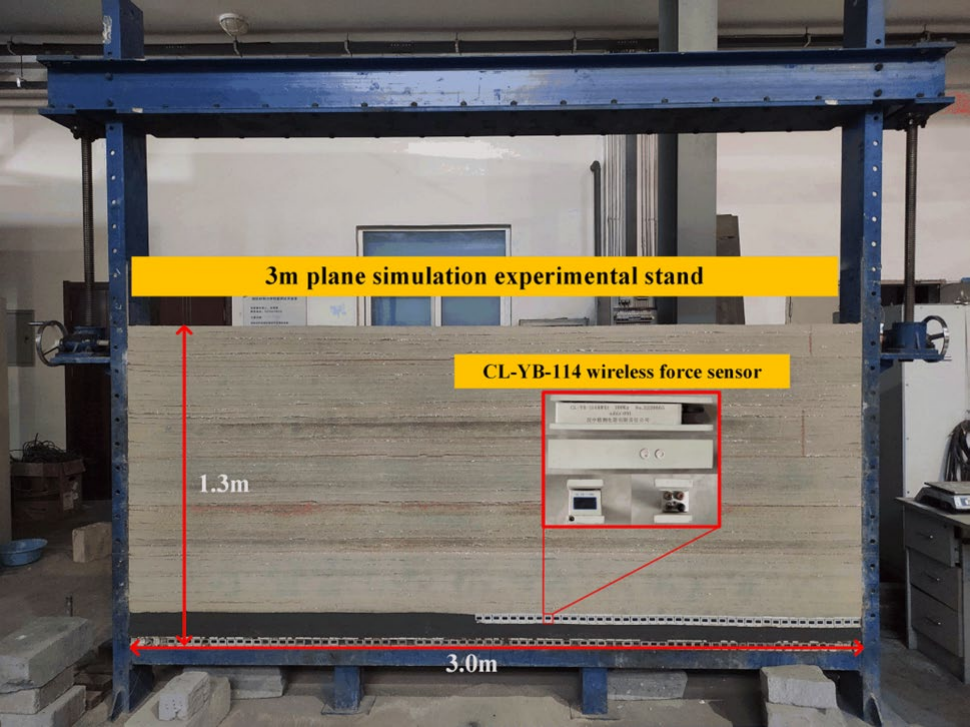
Physical similarity simulation experimental stand.

Choose a 3 m plane simulation test frame. The simulation test frame size: length × width × height = 3000 × 200 × 1800 mm. The equipment needed for the experiment are data acquisition system, notebook computer and digital camera. The data acquisition system is divided into CL-YB-114 pressure sensor and 108 pressure computers, which are used to monitor the upper and lower boundary stress of top-coal. The size of the pressure sensor is: length × width × height = 20 cm × 3.5 cm × 4 cm, with the measuring range of 200 kg. The notebook computer is used to store stress data and process experimental pictures, and the digital camera is used to record the caving patterns of overlying strata in different periods, as shown in Fig. 3. See Table 2 for the physical similarity constants. See Table 3 for specific similar ratios of coal and rock strata. The physical similarity simulation experiment is shown in Fig. 4.

**Table 2:** Physical similarity constant.

Geometric similarity ratio	Density similarity parameter	Stress similarity parameter	Load similarity parameter	Time similarity parameter
1:100	1.6	160	1.6 × 10^6^	10

**Table 3:** Model’s similar material ratio and laying thickness.

Number	Lithology	Thickness (m)	Model layer thickness (cm)	Ratio (river sand/gypsum/lime powder/coal dust)
1	Coal	9	9	20 1 2 20
2	Carbon mudstone	4	4	8 2 8
3	Mudstone	6	5	8 3 7
4	Fine sandstone	10	6	7 3 7
5	Coarse sandstone	8	8	8 4 6
6	Sandstone	12	12	7 2 7
7	Fine sandstone	20	20	7 4 6
8	Conglomerate	32	32	8 4 6
9	Coarse sandstone	29	29	8 5 5

The specific experimental process is as follows:

1. Boundary coal pillars. In order to eliminate the boundary effect, 30 m boundary coal pillars are set on both sides of the model.
2. Setup entry. Setup entry at a distance of 30 m from the boundary on the left side of the model, with a height of 3 m and a width of 7 m. After the setup entry is arranged, start the work of advancing and caving coal in the working face.
3. Face mining and caving. The mining height is 3 m, and the caving height is 6 m. The advancing distance in the experiment is in strict accordance with the actual advancing step of the 1123 working face in the engineering background, and it is circularly advanced from the left side to the right side of the model according to the circulating footage of 0.8 m.

### The caving state of overlying strata

**Figure 5:**
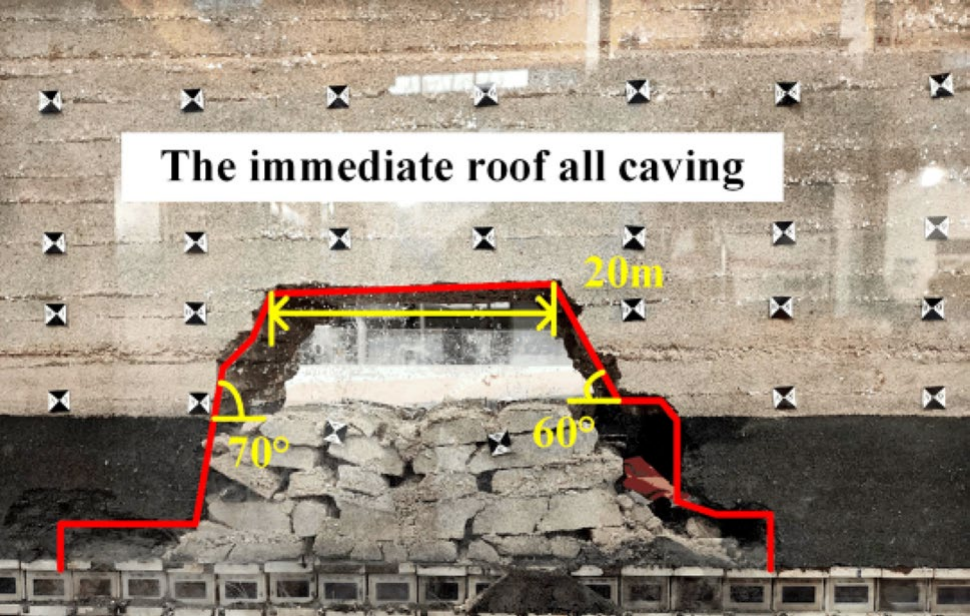
Caving state when advancing to 40 m.

**Figure 6:**
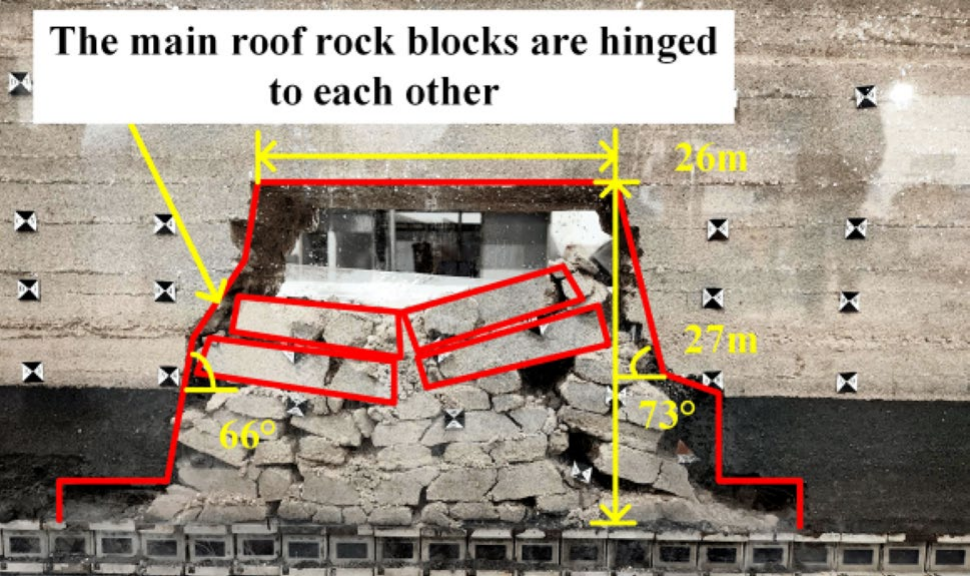
Caving state when advancing to 50 m.

**Figure 7:**
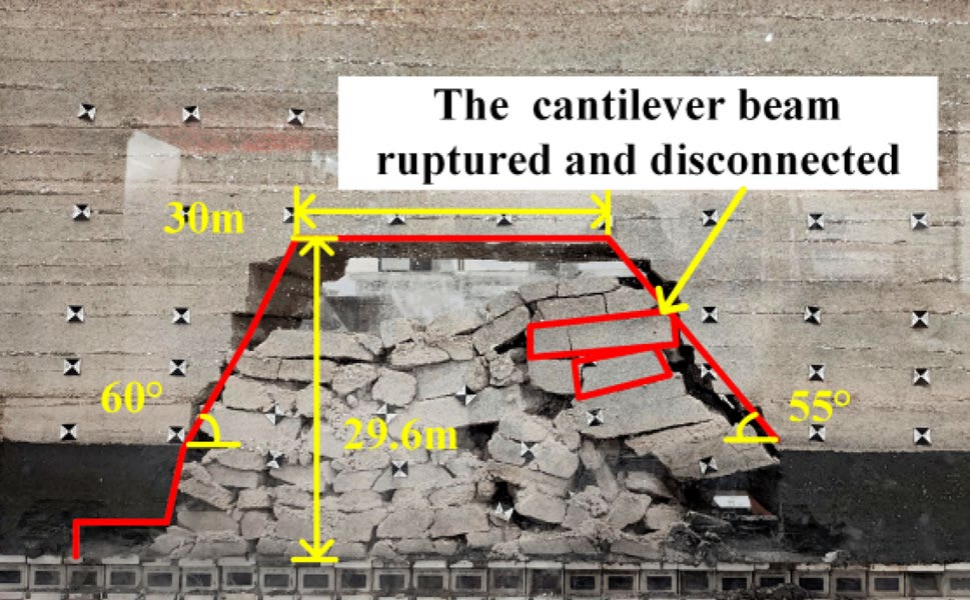
Caving state when advancing to 65 m.

**Figure 8:**
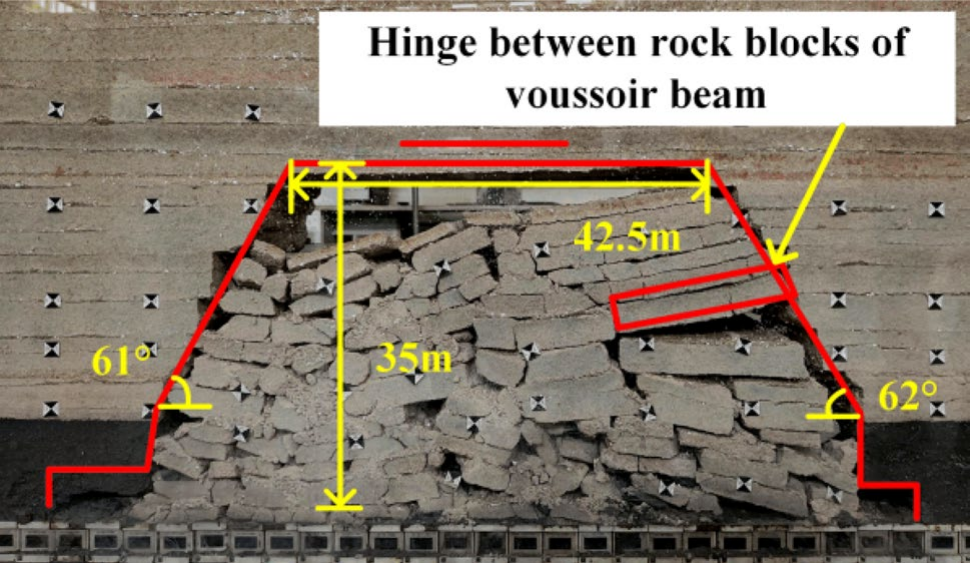
Caving state when advancing to 81 m.

When the working face was advanced to 40 m, the immediate roof completely caving, and the exposed length was 20 m, as shown in Fig. 5. When the working face was advanced to 50 m, the first weighting of the basic roof occurs, with the first weighting interval of 50 m and the exposed length of the roof of 25 m, as shown in Fig. 6. When the working face was advanced to 65 m, the basic roof was deformed and broken in the form of a cantilever beam, and the basic roof was subjected to the first periodic weighting with a weighting step of 15 m, as shown in Fig. 7. When the working face was advanced to 81 m, the basic roof strata are hinged with each other in the form of voussoir beam, and the second periodic weighting of the basic roof occurs, as shown in Fig. 8, with a weighting step of 16 m.

**Figure 9:**
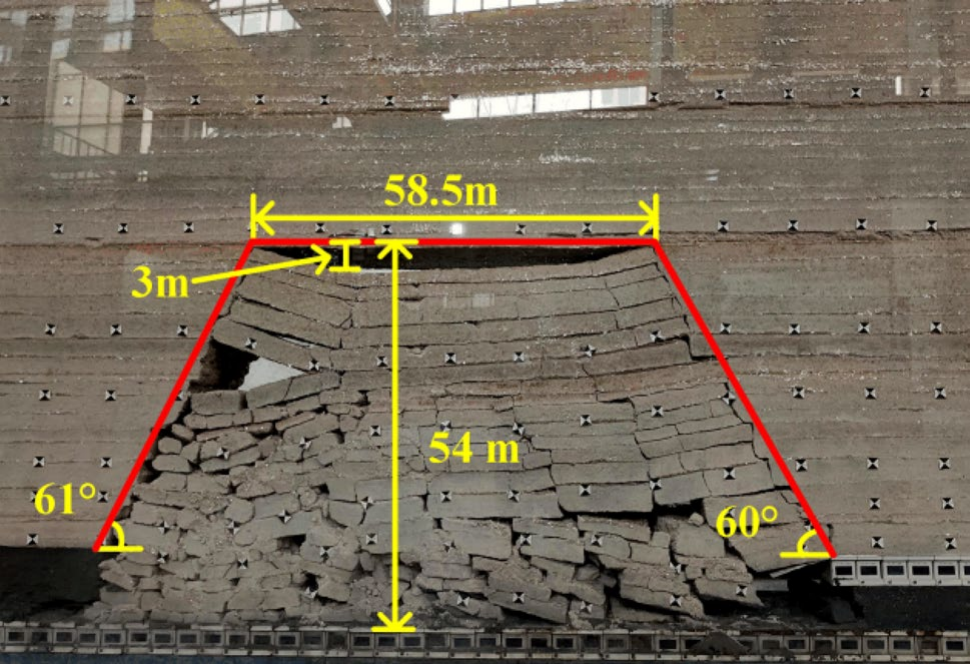
Caving state when advancing to 110 m.

**Figure 10:**
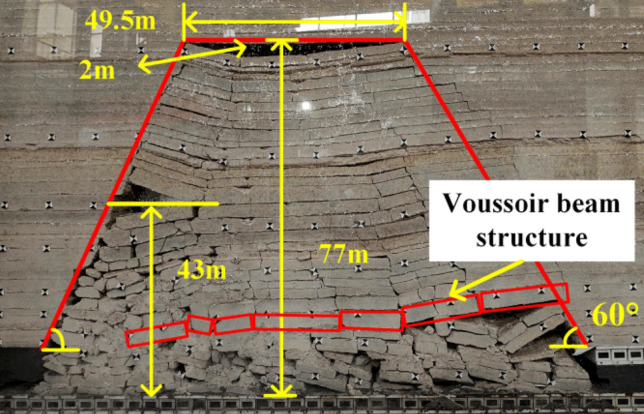
Caving state when advancing to 125 m.

When the working face was advanced to 110 m, the fourth periodic weighting of the main roof occurs, as shown in Fig. 9, the weighting step was 14 m, the exposed length of the roof was 58.5 m, the exposed height was 54 m. At this time, the goaf was basically compacted without obvious gaps. When the working face was advanced to 125 m, the fifth periodic weighting occurs on the main roof, with a weighting step of 15 m. As shown in Fig. 10, the caving zone rises to 43 m, the exposed length of the roof was 49.5 m, the exposed height was 77 m, and the main roof was hinged with each other in a “voussoir beam” structure as a whole.

At the beginning of mining, the caving gangue in goaf did not contact with the overlying strata. With the continuous advancement of working face, the caving arch in goaf continued to expand and the concentrated stress on the top-coal in front of working face continued to increase. When the working face was advanced to 81 m, the gangue in the goaf contacts with the overlying strata. When the working face was advanced to 110 m, the gangue in the goaf was basically compacted, and the strata will be supported again, forming a stable “wall-gangue in the goaf” support system, and the mining-induced stress around the goaf was basically stable^26^. By arranging pressure sensor on the upper boundary of coal seam, the stress data of top-coal in front of working face under different advancing distances were monitored, and the vertical stress distribution law of the upper boundary of top-coal was analyzed.

### Distribution law of top-coal boundary stress

According to the caving characteristics of overlying strata, the working face was advanced to 110 m and the mining-induced stress was stable. The upper and lower boundary stress data of top-coal when the working face was advanced to 110 m, 125 m, 140 m, 157 m, 170 m and 185 m were selected respectively.

**Figure 11:**
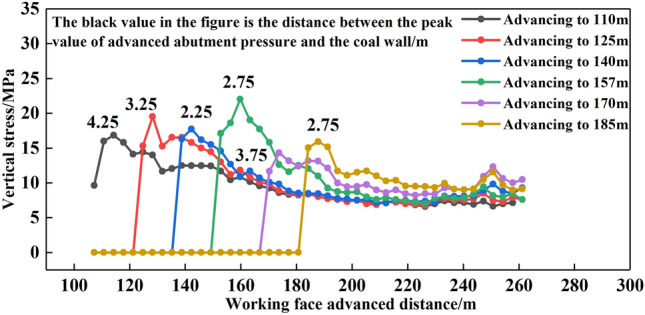
Vertical stress distribution law at the upper boundary of top-coal.

The distribution law of vertical stress at the upper boundary of top-coal was shown in Fig. 11. When the working face was advanced to 110 m, the front supporting pressure peak position of the upper boundary of top-coal was 4.25 m away from the langwall face, and the peak stress was 16.84 MPa. When the working face was advanced to 125 m, 140 m, 157 m, 170 m and 185 m respectively, the peak values of front abutment pressure were 19.54 MPa, 17.75 MPa, 22.18 MPa, 14.34 MPa and 15.94 MPa respectively, and the distances between the peak values position and the langwall face were 2.25 m, 2.75 m, 3.75 m and 2.75 m respectively.

**Figure 12:**
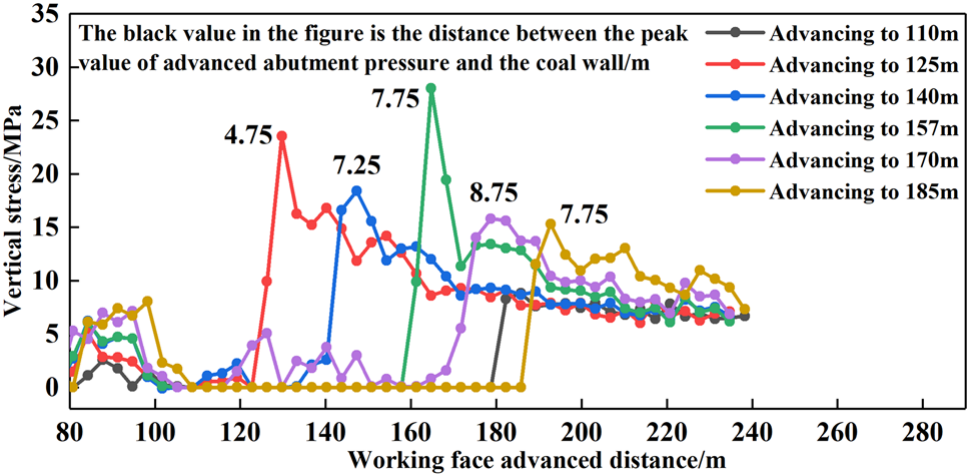
Vertical stress distribution law at the lower boundary of top-coal.

The distribution law of vertical stress at the lower boundary of top-coal was shown in Fig. 12. When the working face was advanced to 125 m, the peak value of front abutment pressure was 23.52 MPa, and the peak position was 4.75 m away from the langwall face. When the working face was advanced to 140 m, 157 m, 170 m and 185 m respectively, the peak values of front abutment pressure were 18.38 MPa, 28.00 MPa, 15.80 MPa and 15.30 MPa respectively, and the distances between the peak positions and the langwall face were 7.25 m, 7.75 m, 8.75 m and 7.75 m respectively.

With the compaction of the goaf behind the working face, the vertical stress was stable, and the distance between the peak position of front abutment pressure on the upper boundary of top-coal and the langwall face was maintained at about 3 m, and the distance between the peak position of front abutment pressure on the lower boundary of top-coal and the langwall face was maintained at about 7 m, and the range of front abutment pressure was maintained at about 50 m.

## Boundary of the top-coal limit equilibrium zone along the strike direction of working face

**Figure 13:**
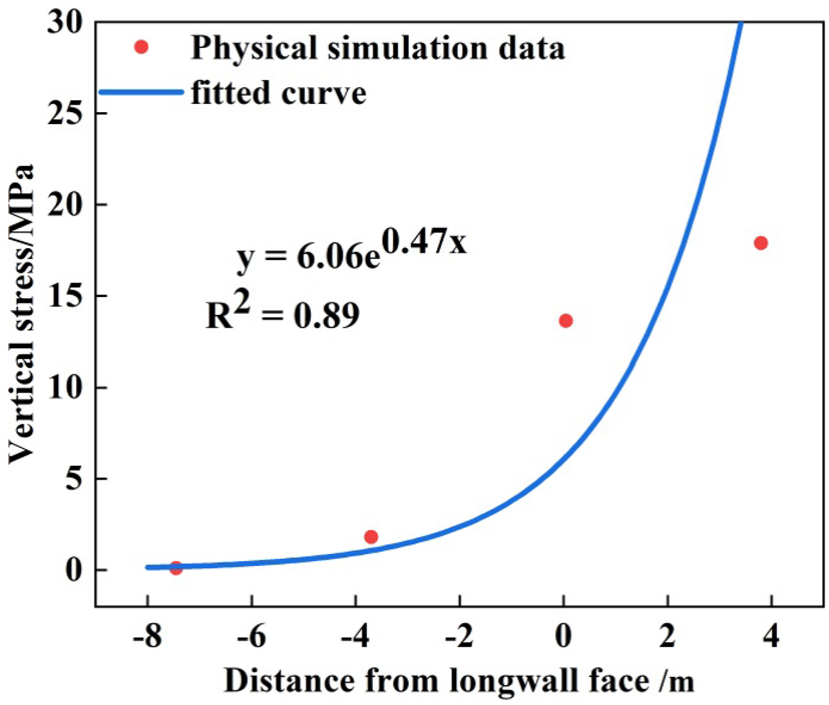
Curve fitting of vertical stress law at the upper boundary of top-coal.

Through the physical similarity simulation experiment, the distribution law of the upper boundary stress of the top-coal was obtained after the mining-induced stress of the working face was stable. Extract the stress data from the vertical stress peak to the langwall face in Fig. 10 and average the data. The scattered distribution characteristics of vertical stress on the upper boundary of top-coal were obtained, as shown in Fig. 13. It can be seen that the scattered data was exponential, and the blue fitting curve was obtained by curve fitting. The curve fitting degree was 0.89, and the fitting curve shows exponential distribution law.

A functional relation about the variation of vertical stress at the upper boundary of top-coal with *x* was obtained:

12 $$\sigma_{y} = 6.06e^{0.47x}$$

By substituting Eq. (12) into Eq. (11), the undetermined constant in Eq. (11) can be determined:

13 $$\left\{ \begin{gathered} W^{\prime}_{1} \;W^{\prime}_{2} { = 6}{\text{.06e}}^{ - 0.47m\tan \varphi } \hfill \\ W = 0.47\tan \varphi \hfill \\ W_{1} = 0 \hfill \\ \end{gathered} \right.$$

Substituting the constants in Eq. (13) into Eq. (11) can obtain the functional relationship between vertical stress and coordinates *x* and *y*:

14 $$\sigma_{y} = 6.06e^{{ - 0.47{\text{m}}\tan \varphi }} e^{{\left[ {\frac{0.47\tan \varphi (x + y\tan \varphi )}{{\tan \varphi }}} \right]}}$$

Substitute Eq. (14) into Eq. (1):

15 $$\tau_{xy} = - \left( {6.06e^{{ - 0.47{\text{m}}\;{\text{t}}an\varphi }} e^{{\frac{0.47\tan \varphi (x + y\tan \varphi )}{{\tan \varphi }}}} \tan \varphi + c} \right)$$

Due to the stress balance of the coal in the top-coal limit equilibrium zone, the resultant force in the X direction in the top-coal limit equilibrium zone in Fig. 2 was 0:

16 $$m\lambda \sigma_{y} (x_{0} ,\;m) + 2\int_{0}^{{x_{0} }} {\tau_{xy} } dx = 0$$

17 $$m\lambda \frac{{d\sigma_{y} (x_{0} ,\;m)}}{dx} - 2\tau_{xy} = 0$$

18 $$x + y\tan \varphi = \ln \left( {\left| { - \frac{2c\tan \varphi }{{2.85\tan \varphi mAe^{{ - 0.47{\text{m}}\tan \varphi }} + 12.12e^{{ - 0.47{\text{m}}\tan \varphi }} \tan^{2} \varphi }}} \right|} \right)0.47$$

The equation of the boundary position of top-coal in fully mechanized top-coal caving face along the strike direction of working face was obtained.

19 $$x = \ln \left( {\left| { - \frac{2c\tan \varphi }{{2.85\tan \varphi mAe^{{ - 0.47{\text{m}}\tan \varphi }} + 12.12e^{{ - 0.47{\text{m}}\tan \varphi }} \tan^{2} \varphi }}} \right|} \right)0.47 - y\tan \varphi$$

**Figure 14:**
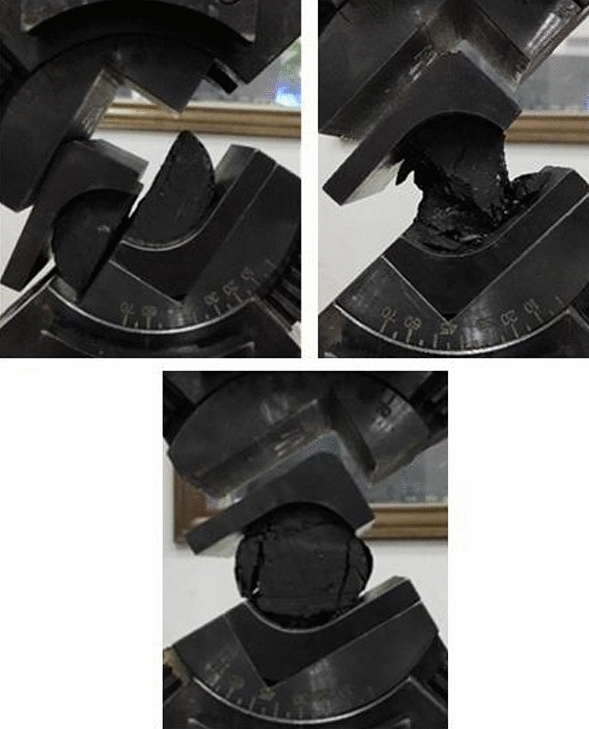
Shear experiment of coal sample.

**Figure 15:**
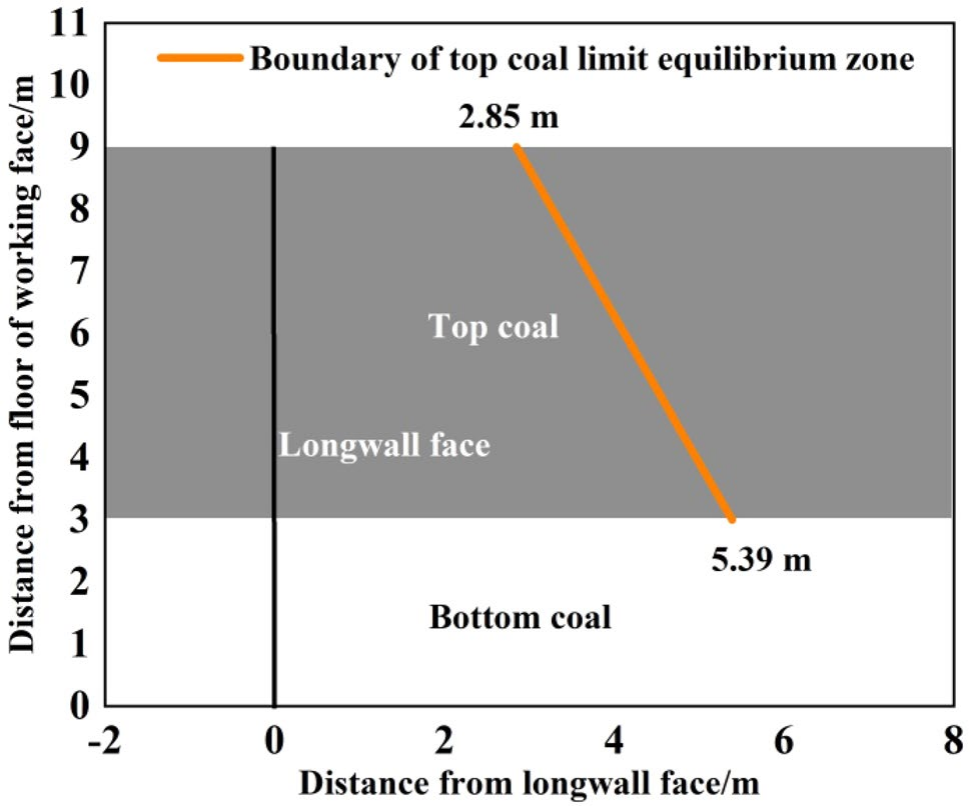
Boundary of the top-coal limit equilibrium zone along the strike direction of working face.

In Eq. (19), the unknown parameters included top-coal thickness m, cohesion *c*, internal friction angle *φ* and coefficient of lateral pressure A. The top-coal thickness was 6 m according to the engineering background. The coefficient of lateral pressure can be determined as 0.70 according to the regression formula of the ratio of average horizontal principal stress to vertical stress^27^. The cohesion and internal friction angle of coal were determined to be 2.7 MPa and 23° respectively through shear experiments, as shown in Fig. 14. The parameters were brought into Eq. (19), and the position relationship of the boundary of the top-coal limit equilibrium zone of fully mechanized top-coal caving face along the strike direction of the coal face was obtained, as shown in Fig. 15.

The orange line was the boundary of the top-coal limit equilibrium zone. The top-coal before the boundary line was in the elastic zone, and no damage has occurred. After the boundary line, under the mining-induced stress, meso-cracks in coal body originated and developed. Near the peak of the front abutment pressure, the development scale and density of cracks began to affect the macroscopic strength of top-coal. At this moment, the macro-cracks of top-coal formed, the damage accumulation began to accelerate, and the elastic state changed to the limit equilibrium state.

When the mining-induced stress of the working face was stable, the upper boundary of the top-coal was closest to the langwall face, with a distance of 2.85 m, and the lower boundary of the top-coal was farthest from the langwall face, with a distance of 5.39 m. Along the strike direction of working face, the distance between the boundary of top-coal limit equilibrium zone and langwall face presents an approximate linear relationship that increases gradually from top to bottom.

## Boundary verification of the top-coal limit equilibrium zone

Through theoretical analysis and physical similarity simulation experiments, the equation of the boundary of the top-coal limit equilibrium zone along the strike direction of working face was derived, and the boundary shape of the top-coal limit equilibrium zone along the strike direction of working face was revealed. In order to verify the authenticity of the results, UDEC numerical simulation experiment was selected for verification.

### UDEC numerical model

**Figure 16:**
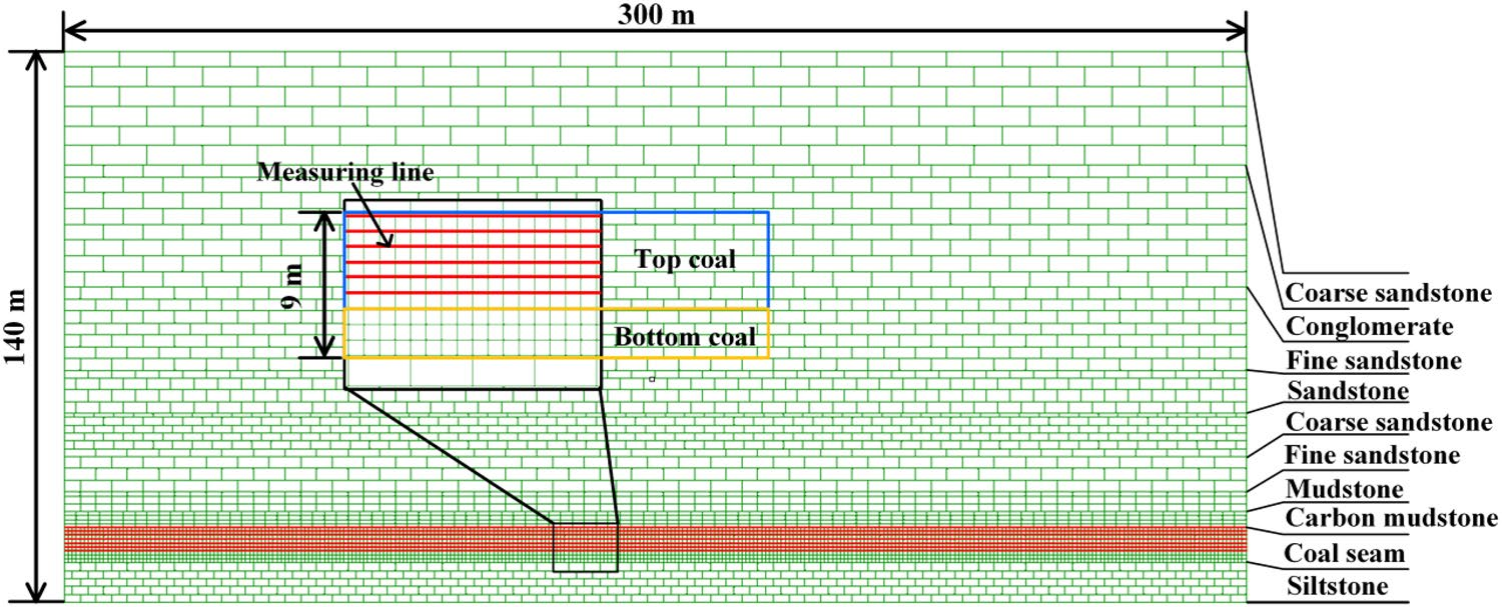
UDEC numerical calculation model.

Based on the engineering background of 1123 working face, a numerical model was established, the length and height of which are 300 m and 140 m respectively. Different rock characteristics divide different joint surfaces, and with the gradual decrease of rock buried depth, the block division becomes larger. As shown in Fig. 16, the model is divided into 10 layers with 21,452 blocks. The left and right side of that numerical model are respectively provided with 30 m boundary coal pillar. The vertical pressure of 5 MPa is applied to the upper boundary of the model to simulate the load layer of 200 m. Gradient stress is applied to the left and right boundaries respectively, and the upper boundary and the left and right boundaries restrict the movement of the model respectively. Mohr–Coulomb model is used for model blocks, and Coulomb sliding model of surface contact is selected for joint constitutive model.

The parameter cohesion and internal friction angle in the numerical model were selected by the method proposed by Kazerani^28^, and the tensile strength was usually considered to be one-tenth of the uniaxial compressive strength^29^. According to Wilson’s suggestion^30^, the modulus of elasticity in GPa was 0.31 times of the uniaxial compressive strength in MPa. The bulk modulus, shear modulus, normal stiffness and shear stiffness in the numerical model were determined by the calculation method of UDEC numerical simulation software, from which the aforementioned parameters for each stratum in the model can be determined.

The numerical simulation experiment steps are as follows:

1. Arrange survey lines in coal seam, with a total of 7 survey lines.
2. The process of mining and caving coal in working face. First, setup entry is carried out, and the model simulates setup entry by deleting blocks, when the model reaches balance, the coal seam is mined with a mining height of 3 m. After mining, the top-coal is removed to recover the coal seam. The caving height is 6 m and the mining-caving ratio is 1:2. After the mining and coal caving work is completed, the mining and caving work will be repeated in the next cycle, and each cycle will be advanced by 1 m until the end of mining. The loop is completed by the built-in programming language (FISH) in the UDEC model.
3. Analyze the results.

### Boundary of top-coal shear failure under different advancing distances

**Figure 17:**
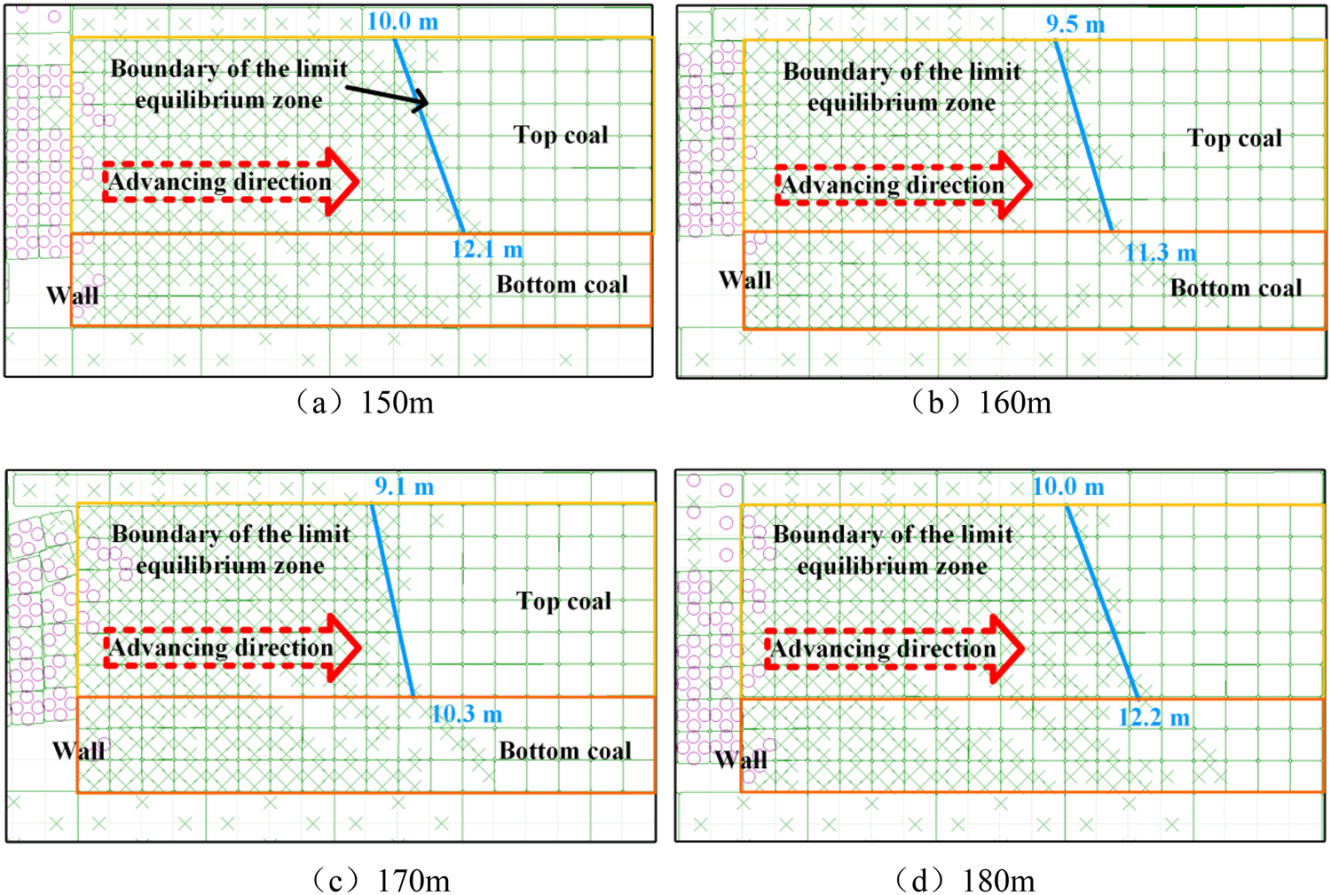
Boundary of top-coal shear failure under different advancing distances.

When the mining-induced stress in front of the langwall face was stable. The position where shear failure of the top-coal begins to occur intensively was considered the boundary of the limit equilibrium zone^24^. The boundary shape of plastic zone of top-coal under different advancing distances was selected to verify the rationality of the boundary of limit equilibrium zone of top-coal along the strike direction of working face. Select the plastic zone of top-coal in front of the coal when the advancing distance was 150 m, 160 m, 170 m and 180 m respectively, as shown in Fig. 17. The coal near the langwall face was in a state of tensile failure. With the extension to the advancing direction, the tensile failure of coal turns into shear failure, which continues to extend to the deep, and the shear failure area was sparse, and the coal gradually returns to the elastic state. It can be seen from Fig. 17a–d that the shear failure zone of the top-coal was regular trapezoid. The distance between the elastic–plastic boundary and the langwall face was an approximate linear relationship that gradually increases from top to bottom along the strike direction of working face. When the working face advances to 150 m, 160 m, 170 m and 180 m, the horizontal distances between the upper boundary of the elastic–plastic boundary of the top-coal and the langwall face were 10.0 m, 9.5 m, 9.1 m and 10.0 m respectively. The horizontal distances between the lower boundary of the top-coal elastic–plastic boundary and the langwall face were 12.1 m, 11.3 m, 10.3 m and 12.2 m respectively.

### Peak boundary of front abutment pressure of top-coal under different advancing distances

When the top-coal in front of the working face is near the peak value of front abutment pressure, the bearing capacity of coal seam reaches the limit. Top-coal starts to extend on the basis of original cracks and joints until they are interconnected. The deterioration degree of top-coal failure continues to intensify until it becomes loose block. Therefore, the boundary of top-coal limit equilibrium zone can be indirectly verified by the peak position of front abutment pressure.

**Figure 18:**
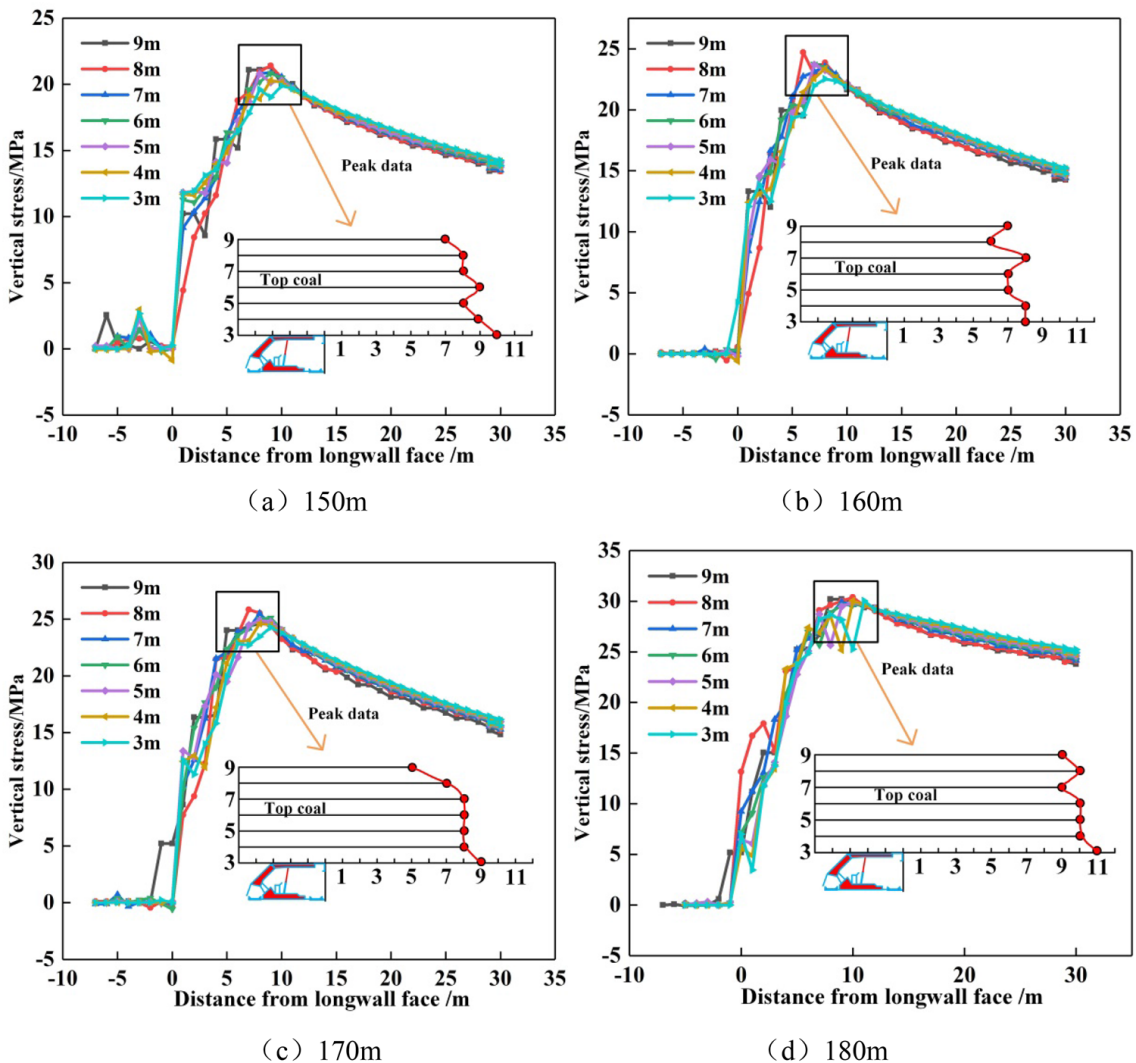
Peak boundary of front abutment pressure of top-coal under different advancing distances.

By extracting the peak of the front abutment pressure at different heights from the top-coal to the floor and connecting the peak positions, as shown in the lower right corner of Fig. 18. When the working face advances to 150 m, the distance between the peak value of front abutment pressure at 9 m to 3 m of coal seam and the langwall face was 7 m, 8 m, 8 m, 9 m, 8 m, 9 m and 10 m respectively. When the working face advances to 160 m, the distance between the peak value of front abutment pressure at 9 m to 3 m of coal seam and the langwall face was 7 m, 6 m, 8 m, 7 m, 7 m, 8 m and 8 m respectively. When the working face advances to 170 m, the distance between the peak value of front abutment pressure at 9 m to 3 m of coal seam and the langwall face was 5 m, 7 m, 8 m, 8 m, 8 m, 8 m and 9 m respectively. When the working face advances to 180 m, the distance between the peak value of front abutment pressure at 9 m to 3 m of coal seam and the langwall face was 9 m, 10 m, 9 m, 10 m, 10 m, 10 m and 11 m respectively. It can be seen from the boundary of the peak value of front abutment pressure with different advancing distances in the working face that the peak value of front abutment pressure was farther and farther away from the langwall face of the working face as the distance between the top-coal and the floor gets closer.

## Discussion

**Figure 19:**
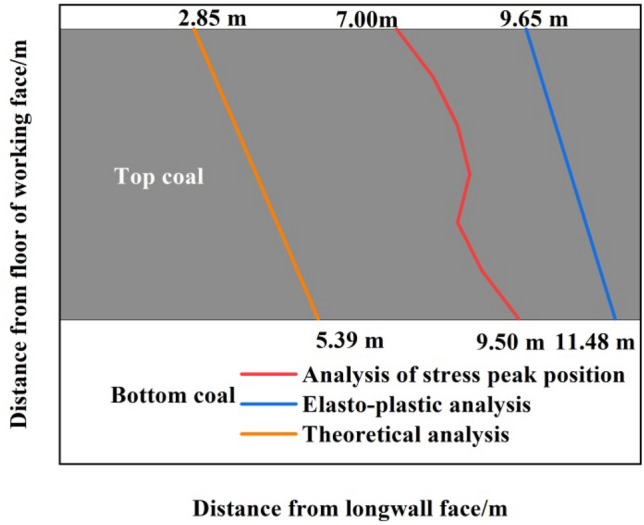
Comparative analysis of theoretical calculation and numerical calculation.

This paper determined the boundary of top-coal limit equilibrium zone along the strike direction of working face by combining theory with experiment. Along the strike direction of working face, the distance between the boundary of top-coal limit equilibrium zone and longwall face showed an approximate linear relationship of increasing from top to bottom. Combined with UDEC numerical simulation experiments, the boundary of elastic–plastic zone of top-coal and the boundary of peak position of front abutment pressure in different strata of top-coal were obtained respectively. A comparison chart of the results as shown in Fig. 19 was obtained. Many results show that the distance between the boundary and the longwall face along the strike direction of working face is increasing from top to bottom. Relevant scholar determined the shear failure zone through numerical simulation and crack propagation mechanism when studying the stress-driven mechanism of crack propagation in top-coal caving mining^13^. The boundary of shear failure zone was very consistent with the boundary of top-coal limit equilibrium zone along the strike direction of working face studied in this paper.

The quantitative characterization of the boundary of top-coal limit equilibrium zone is a general solution by combining the stress state characterization of top-coal with Mohr–Coulomb criterion. Combined with the mining-induced stress of actual working conditions, the correlation coefficient is determined, and the boundary of top-coal limit equilibrium zone is finally quantified. A new method of the position relationship between the boundary of the top-coal limit equilibrium zone and the longwall face along the strike direction of the working face is established. The advantage of this method is a wide range of application. For fully mechanized top-coal caving faces under different conditions, the position relationship between the boundary of the top-coal limit equilibrium zone and the longwall face along the strike direction of the working face can be determined. However, before determining the position relationship, it is necessary to fully study the mining-induced stress and determine the detailed physical and mechanical parameters of coal seam. This is also the disadvantage of this method, that is, the research time is longer. In the future, the fully mechanized top-coal caving face under different working conditions will be studied and analyzed, and the method of determining the boundary of top-coal limit equilibrium zone will be further optimized through deep learning. Aiming at the two extreme engineering situations of hard coal seam and soft coal seam, this paper puts forward some targeted measures in combination with the research content of this paper.

**Figure 20:**
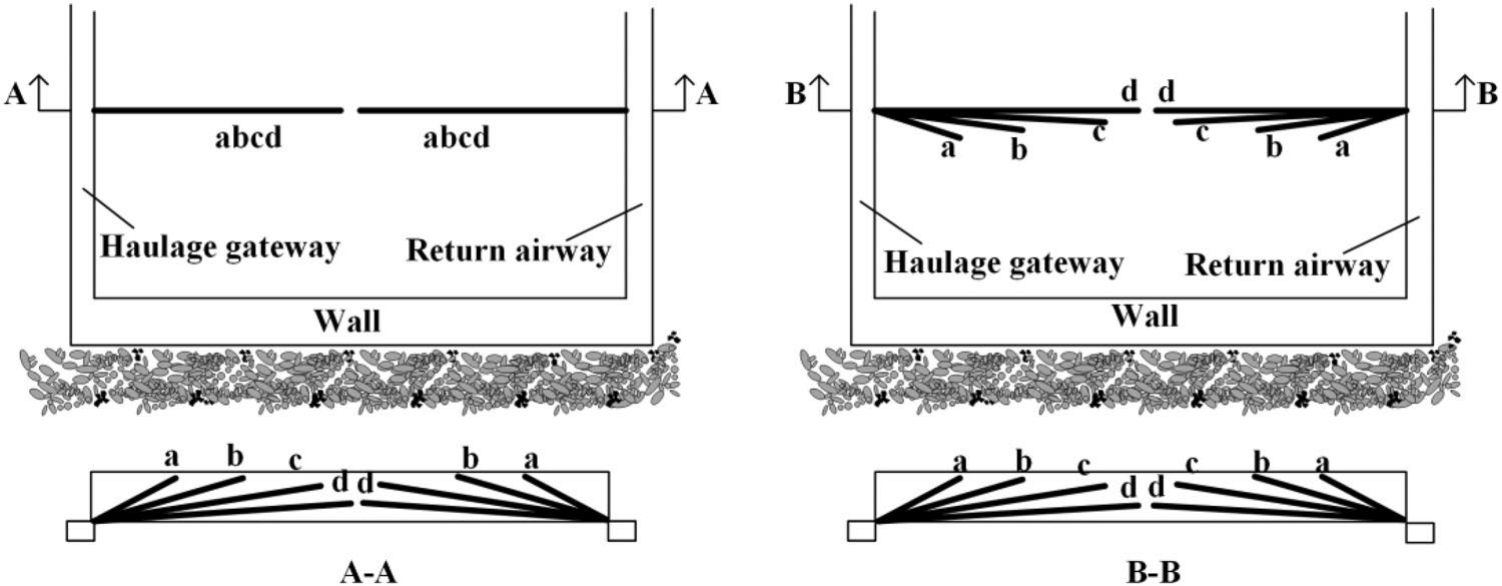
Comparison of pre-splitting process of hard top-coal.

For the working face of hard coal seam, due to the high strength of coal seam, the coal seam is not fully broken under the mining-induced stress. The coal caving process is blocked, which seriously affects the production efficiency. Generally, the top-coal is pre-cracked, and the pre-splitting techniques include blasting pre-splitting^31,32^ and hydraulic fracturing^33^. The general drilling construction layout is shown on the left side of Fig. 20. Boreholes along the strike direction of the working face are usually arranged vertically. Combined with the research results of this paper, the drilling layout direction can be improved. The vertically arranged boreholes are changed to be arranged along the boundary of the limit equilibrium zone, as shown on the right side of Fig. 20. Under this arrangement, the peak position of mining-induced stress coincides with the boundary position of pre-splitting coal body. The superposition effect is achieved, so that the top-coal is crushed more evenly and the crushing efficiency is higher, the caving rate is improved, and the production efficiency of the coal mine is guaranteed.

For the working face of soft coal seam, under the action of mining-induced stress, the coal seam has been fully broken before reaching the working face. It leads to partial roof fall, which seriously affects workers' safety and mine production efficiency. Combined with the research results of this paper, the targeted measures are put forward:

1. When the double drum shearer is mined, the front beam of the hydraulic powered support should be extended in time. Put the front beam close to the longwall face or insert it into the coal body to reduce the possibility of partial roof fall as much as possible.
2. Broken coal is easy to leak between supports when passing above them. Therefore, it is necessary to lay steel wire mesh above the support to ensure the safety of workers as much as possible.
3. According to the weak coal seam, the mining-caving ratio can be adjusted appropriately. For example, reduce the mining height of the working face and increase the caving height. According to Eq. (19), the caving height is inversely proportional to the distance between the top-coal limit equilibrium zone and the longwall face. When the boundary of the limit equilibrium zone is closer to the longwall face, the degree of top-coal fragmentation will be less when it reaches the working face, thus achieving the purpose of slowing down the degree of top-coal fragmentation.

## Conclusion

1. Based on theoretical analysis, the stress state characterization method of the boundary of top-coal limit equilibrium zone along the strike direction of working face was given. Combined with mining-induced stress, the quantitative characterization of the boundary of top-coal limit equilibrium zone along the strike direction of working face was realized. The spatial relationship between the boundary of the top-coal limit equilibrium zone and the longwall face along the strike direction of working face was revealed. The distance from the boundary of the top-coal limit equilibrium zone along the strike direction of working face to the longwall face gradually increased from top to bottom.
2. Taking 1123 fully mechanized top-coal caving face as the research object, the boundary position of top-coal limit equilibrium zone was compared and verified from two aspects of failure form and stress distribution through numerical calculation. The results show that the new method to determine the position relationship between the boundary of the top-coal limit equilibrium zone along the strike direction of working face and the longwall face was reasonable and reliable. According to the research results, targeted measures were put forward for hard coal seam and weak coal seam to improve mine efficiency.

## Data Availability

The data that support the findings of this study are available from the corresponding author upon reasonable request.
